# Implications of pseudogenes for the prognosis of hepatocellular carcinoma

**DOI:** 10.1002/ctm2.1195

**Published:** 2023-02-07

**Authors:** Zaoqu Liu, Yuyuan Zhang, Siyuan Weng, Hui Xu, Xinwei Han

**Affiliations:** ^1^ Department of Interventional Radiology The First Affiliated Hospital of Zhengzhou University Zhengzhou China; ^2^ Interventional Institute of Zhengzhou University Zhengzhou China; ^3^ Interventional Treatment and Clinical Research Center of Henan Province Zhengzhou China

Dear Editor

Growing evidence has demonstrated that pseudogenes are intricately associated with tumour progression in hepatocellular carcinoma (HCC).^1^ Nonetheless, pseudogenes related to tumour immune infiltration and their value in improving clinical outcomes remain largely unexplored. Pseudogenes, newly discovered non‐coding homologs of protein‐coding genes,^1^ have been regarded as non‐functional evolutional relics. Nevertheless, increasing evidence has demonstrated that pseudogenes play vital roles in tumourigenesis as regulators of coding genes. For example, *HMGA1P6*, a pseudogene transcriptionally activated by oncogene *MYC*, could contribute to oncogenesis in ovarian cancer.^2^ Besides, pseudogenes also have profound impacts on anti‐tumour responses via involvement in regulating tumour‐immune interactions. *BRCA1* Pseudogene 1 (*BRCA1P1*) has been reported to weaken immune response by suppressing innate immune defense mechanisms.^3^ Through glioma‐derived exosomes, *TMEM198B* promoted macrophage lipid accumulation and increased fatty acid oxidation, further inducing macrophages to M2 polarization.^4^ However, the clinical significance of immune‐related pseudogenes remains largely unexplored in HCC.

Here, we enrolled four public datasets with abundant pseudogene expression profiles and complete prognostic information, including: The Cancer Genome Atlas Liver Hepatocellular Carcinoma (TCGA‐LIHC) (*n* = 340), GSE116174 (*n* = 64), GSE144269 (*n* = 67) and GSE14520 (*n* = 242) (Table S1). To systematically evaluate candidate intrinsic pseudogene modulators of immune cells, we introduced a novel framework to identify immune‐related housekeeping pseudogenes (Supplementary Materials). In TCGA‐LIHC, the relative abundance of 28 immune cells was first deciphered using the ssGSEA algorithm.^5^ Considering that tumour purity may obscure links in tumour microenvironment,^6^ we thus calculated the first‐order partial correlation coefficient (PCC) between pseudogenes and immune cells by removing the effect of tumour purity.^6^, ^7^ Pseudogenes with the top 5% PCC were extracted as candidate immune‐related pseudogenes for each immune cell. A hypothesis is that if a specific pseudogene has strong correlations with all immune cell types, it may execute a housekeeping role in HCC immune microenvironment,^8^ which is also defined as HCC immune‐related intrinsic pseudogene (HIRIP) in this study. Here, we calculated the tissue specificity index (TSI)^9^ to identify pseudogenes generally correlated with different immune cell types (Table S2). As previously reported,^9^ pseudogenes with a lower TSI score were strongly associated with all immune cell types, suggesting their vital biological functions in immunity. According to the criteria described in previous studies,^8^, ^9^ we settled the threshold of TSI <.2 and identified 23 HIRIP essential for immune regulation (Figure S1A).

Subsequently, the 23 HIRIP were further filtered to develop an integrative HIRIP signature (HIRIPS). Initially, univariate Cox regression analysis determined four pseudogenes with prognostic potential, including *HNRNPA3P5*, *HNRNPA3P6*, *PTMAP5* and *EIF2S2P4* (Figure S1B). Kaplan–Meier analysis demonstrated that high expression of these four pseudogenes suggested an unfavorable prognosis in HCC (Figure 1A). Afterward, to identify an optimal machine‐learning algorithm for assessing prognosis, we developed 22 types of survival machine‐learning models (Supplementary Materials) based on the four pseudogene expression profiles of TCGA‐LIHC. As mentioned above, A final HIRIPS fitted by the Akritas algorithm with the highest average mean C‐index (.667) and integrated areas under the curve (iAUC) (.825) in the TCGA training cohort and three validation cohorts (GSE116174, GSE144269 and GSE14520) was considered the optimal one (Figure 1B).

**FIGURE 1:**
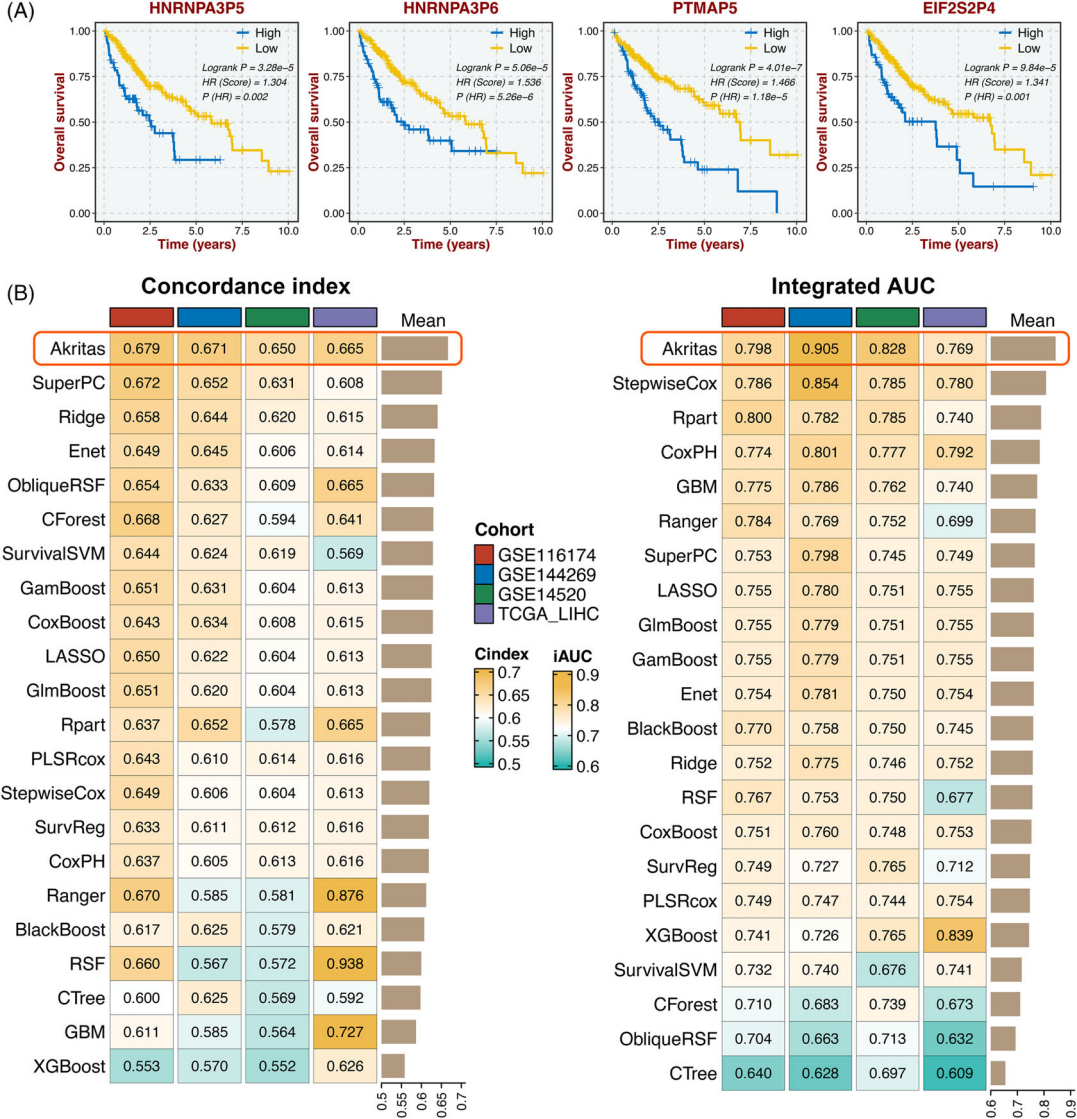
Integrative construction of a consensus signature. (A) Kaplan–Meier analysis of four immune‐related intrinsic pseudogenes in TCGA‐LIHC. (B) C‐indices (left) and iAUC (right) of 22 survival machine‐learning models in GSE116174, GSE144269, GSE14520, and TCGA‐LIHC

To validate the robust performance of HIRIPS, Kaplan‐Meier and Cox regression survival analyses were applied. Notably, patients with high HIRIPS possessed adverse prognosis in TCGA‐LIHC (HR = 1.713; log‐rank test: *P* < .001), and similar tracks were observed in three validation datasets, GSE116174 (HR = 1.136; log‐rank test: *P* < .001), GSE144269 (HR = 1.120; log‐rank test: *P* < .001), and GSE14520 (HR = 1.118; log‐rank test: *P* < .001) (Figure 2A). Subsequently, time‐dependent receiver operating characteristic (ROC) curves for OS (1‐, 2‐, and 3‐year) exhibited excellent discrimination of HIRIPS (Figure 2B) in TCGA‐LIHC (AUC values: .728, .690, and .699), GSE116174 (AUC values: .714, .751, and .700), GSE144269 (AUC values: .703, .704, and .714), and GSE14520 (AUC values: .691, .694, and .702). Multivariate Cox regression analysis was performed and demonstrated HIRIPS remained statistical significance after adjusting confounding factors across all cohorts (Figure 2C). Overall, HIRIPS presented a clear superior performance in predicting prognosis.

**FIGURE 2:**
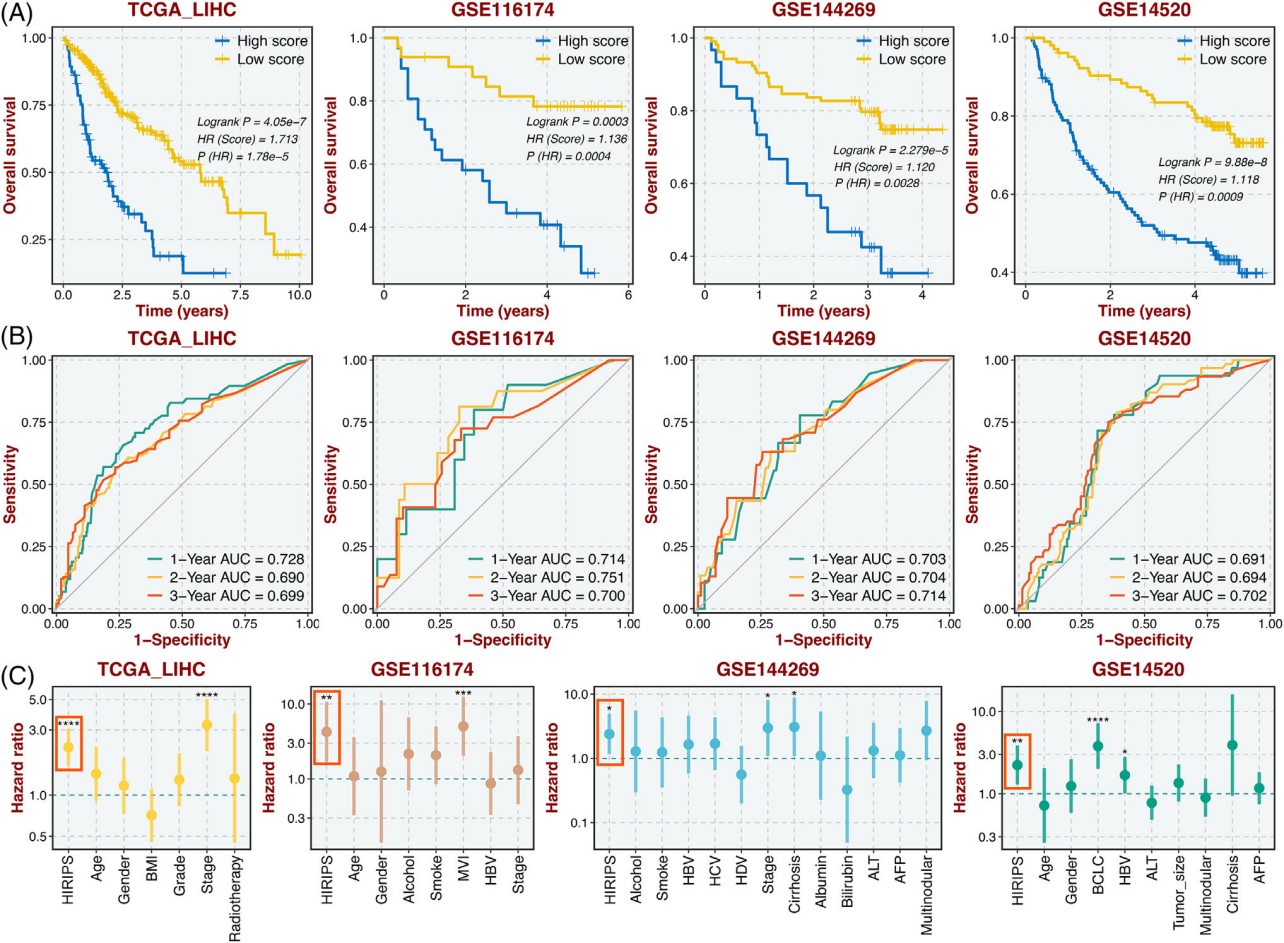
Evaluation of the performance of the HIRIPS model. (A) Kaplan–Meier curves of OS between high and low HIRIPS groups in TCGA‐LIHC, GSE116174, GSE144269, and GSE14520. (B) Time‐dependent ROC analysis for 1, 2 and 3 years in four datasets. (C) Multivariate Cox regression analysis of OS based on HIRIPS and other clinical traits in four datasets. *p < .05; **p < .01; ***p < .001; ****p < .0001

To further explore the biological mechanisms underlying the HIRIPS, we proposed a novel pipeline to perform functional enrichment, which maximized information retention and comprehensively considered gene ordering across all cohorts (Supplementary Materials). The over‐representing analysis revealed patients with high HIRIPS exhibited predominant enrichment in pathways related to malignant progressions, such as cell cycle and p53 signaling (Figure 3A and Figure S2). Whereas patients with low HIRIPS were particularly evident in immune‐related pathways, for example, cytokine activity and T cell receptor pathways (Figure 3A and Figure S2). Subsequently, another bioinformatics algorithm, GSEA, also confirmed that low HIRIPS was significantly linked to immune response (Figure 3B–E). Overall, remarkable differences regarding biological function were identified between the high and low HIRIPS, which may account for the discrepancy in prognosis. Patients with high HIRIPS were featured by cellular proliferation and lower immune status, in line with their unfavorable prognosis. Low HIRIPS was predominantly distinguished by high immune activity and abundant immune cell infiltration, which indicated patients with low HIRIPS harbored more reserves of immunization resources for immunotherapy.

**FIGURE 3:**
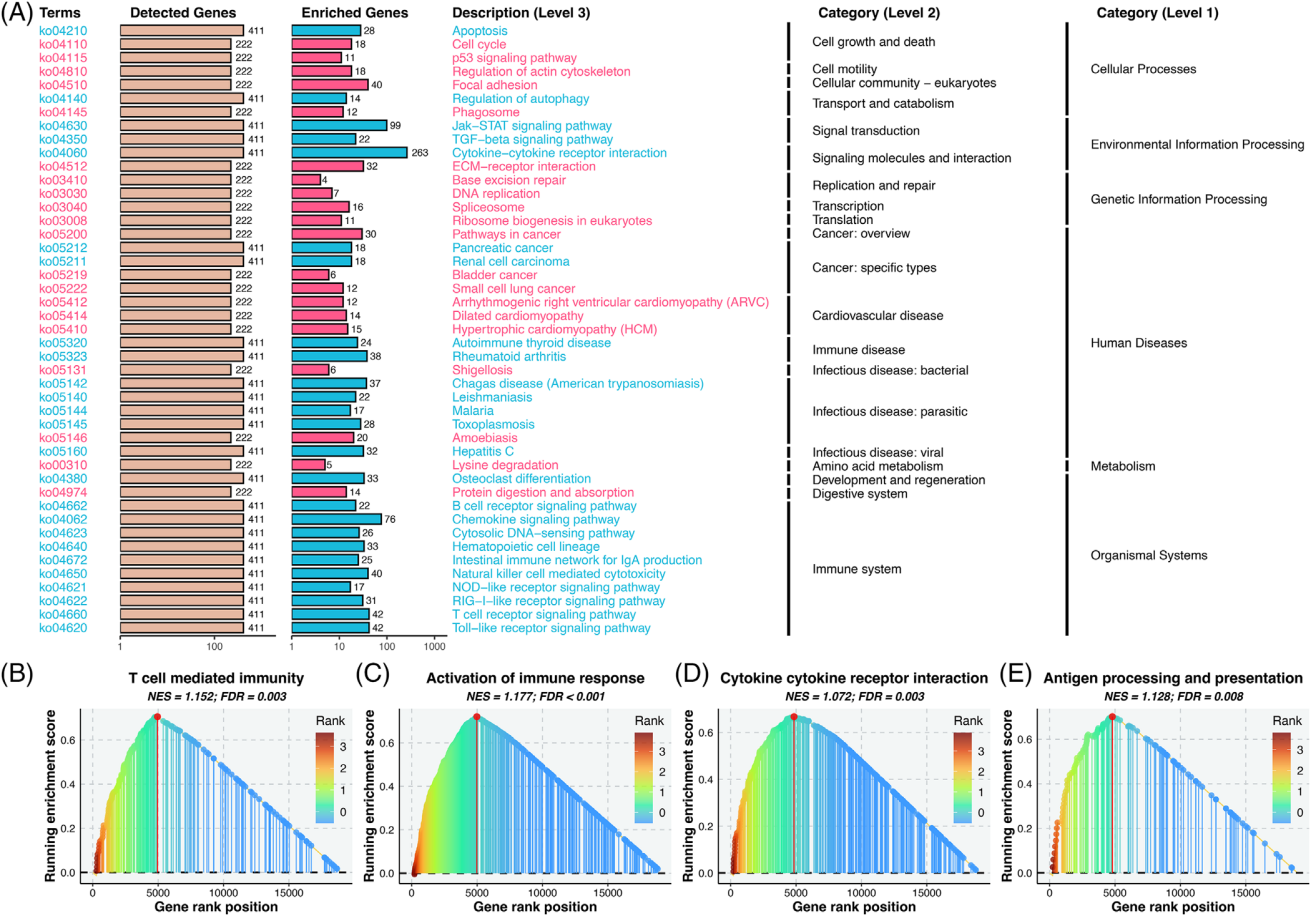
Underlying biological pathways and distinct microenvironment patterns related to HIRIPS. (A) KEGG pathways that were significantly enriched in high HIRIPS are shown in red; those significantly enriched in low HIRIPS are shown in blue. (B–E) Gene set enrichment analysis (GSEA) of immune‐related terms for HIRIPS, including “T cell‐mediated immunity” (B), “activation of immune response” (C), “cytokine cytokine‐receptor interaction” (D) and “antigen processing and presentation” (E)

In conclusion, we comprehensively investigated pseudogenes associated with HCC‐infiltrating immune cells and systematically identified an optimal pseudogene signature (termed HIRIPS) from 22 survival machine‐learning algorithms. This signature displayed a robust and stable performance for predicting prognosis and might also serve as a latent biomarker for assessing immunotherapy response. Overall, our study provided a promising platform for optimizing precise treatment and improving clinical outcomes in HCC.

## CONFLICT OF INTEREST

The authors declare that they have no competing interests.

## Supporting information

Supporting information

Supporting information

Supporting information

Supporting information
