# Supplementary material for: Implications of pseudogenes for the prognosis of hepatocellular carcinoma
Source: Clin Transl Med. 2023 Feb 7;13(2):e1195. doi: 10.1002/ctm2.1195 (PMC9905005; doi:10.1002/ctm2.1195)
Supplement: Supplementary file 1 — Supporting information [file CTM2-13-e1195-s004.docx]

**Materials and methods**

**Publicly available data generation and processing**

***HCC patient cohorts.*** Transcriptome and clinical data of HCC patients were generated from The Cancer Genome Atlas (TCGA, <https://portal.gdc.cancer.gov>) and Gene Expression Omnibus (GEO, <http://www.ncbi.nlm.nih.gov/geo>). We ultimately enrolled four cohorts with abundant pseudogene profiles and complete prognostic information, including TCGA-LIHC (n =340), GSE116174 (n =64), GSE144269 (n =67), and GSE14520 (n =242). Details of data sources are provided in Table S1.

***Data processing.*** Log_2_ transformation was performed on TCGA-LIHC RNA-seq (raw count) data after converting to transcripts per million (TPM). Raw CEL files from Affymetrix® microarrays were processed and normalized using the affy package to obtain robust multiarray averages (RMA). Z-score normalization was conducted to improve the compatibility between sequencing and microarray data. Patients with follow-up >30 days were retained.

**Development of tumor-infiltrating immune cell-associated** **pseudogene signature**

Based on pseudogene profiles and immune cell infiltration in TCGA-LIHC, pseudogenes related to HCC-infiltrating immune cells were identified using the following computational framework:

1. As previously reported, single sample gene set enrichment analysis (ssGSEA)^1^ was conducted to evaluate the infiltration abundance of 28 immune cells in the tumor microenvironment (TME).
2. Tumor purity in bulk HCC samples was quantified by the ESTIMATE algorithm^2^.
3. We calculated the first-order partial correlation coefficient (PCC) between pseudogene *i* and cell *j* by removing the effect of tumor purity *p*:
   1.
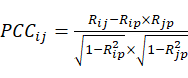

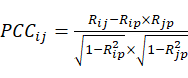

   2. where *R_ij_*, *R_ip_*, and *R_jp_* refer to the Pearson correlation coefficients between pseudogene *i* and cell *j*, pseudogene *i* and tumor purity *p*, as well as cell *j* and tumor purity *p*, respectively.
4. Pseudogenes with the top 5% PCC were extracted as candidate immune-related pseudogenes for each immune cell.
5. The tissue specificity index (TSI) proposed by Yanai et al.^3^ was utilized to assess the specificity of candidate pseudogenes for each cell type:
   1.
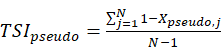

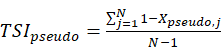

   2. where *N* refers to the total number of immune cell types and *X_pseudo,j_* refers to the relative correlation component normalized by the maximal component value of pseudogene in all immune cell types. Pseudogenes are considered immune-cell-general pseudogenes when TIS is 0, and immune-cell-specific pseudogenes when TIS is 1^4^. Pseudogenes with high PCCs across all cell types were termed HCC immune-related intrinsic pseudogenes (HIRIP). Here, we settled the threshold of TSI <0.2.
6. Pseudogenes with prognostic potential were identified via univariate Cox regression analysis when a *P* value <0.01.
7. To identify an optimal machine-learning algorithm for assessing prognosis, we modified and supplemented the integrative pipeline described in our previous studies^5,6^. We applied 22 types of survival machine-learning algorithms, including Akritas conditional non-parametric survival estimator (Akritas), gradient boosting with regression trees (BlackBoost), conditional random forests (CForest), CoxBoost, proportional hazards Cox regression (CoxPH), conditional inference trees (CTree), elastic net (Enet), gradient boosting for additive model (GamBoost), generalized boosted regression model (GBM), gradient boosting with component-wise linear model (GlmBoost), least absolute shrinkage and selection operator (LASSO), oblique random survival forest (ObliqueRSF), partial least squares regression generalized linear model (PLSRcox), regression for a parametric survival model (Ranger), random survival forest (RSF), regression for a parametric survival model (SurvReg), Ridge, recursive partitioning and regression trees (Rpart), stepwise Cox regression analysis (StepwiseCox), supervised principal components (SuperPC), support vector machine survival analysis (SVM), and partial least squares regression generalized linear models (XGBoost), to develop machine leaning-derived HIRIP signatures (HIRIPS) in TCGA-LIHC.
8. 22 types of HIRIPS models were subsequently validated in GSE116174, GSE144269, and GSE14520. The model possessing the highest mean C-index and integrated AUC (iAUC) was considered the optimal one.
9. Kaplan-Meier survival analysis, time-dependent receiver operating characteristic (ROC) curve analysis, and multivariate Cox regression analysis were utilized to measure the performance of the optimal model.

**Functional enrichment**

Functional enrichment e.g., over-representation analysis (ORA) and gene-set enrichment analysis (GSEA) tend to focus on the leading genes with high ranks in a reference gene list^7^. To maximize information retention and comprehensively consider gene ordering across all cohorts, we proposed a novel pipeline to rank genes:

1. For cohort *α*, we first calculated the Spearman correlation coefficient *R_αβ_* between HIRIPS and mRNA *β*. Each cohort generated a correlation vector with the length of available mRNAs.
2. According to the mRNA correlation lists, the robust rank aggregation (RRA) method was utilized to detect mRNAs that are ranked consistently better than expected under the null hypothesis of uncorrelated inputs. For each cohort *α*, RRA performed mRNA list integration and calculated the *ρ_αβ_* score for each mRNA *β*.
3. Subsequently, we determined the final rank score (rs) across four cohorts as follows:


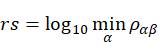


1. The ordered gene list was subjected to Gene Ontology (GO), Kyoto Encyclopedia of Genes and Genomes (KEGG), and GSEA analysis via the *clusterProfiler* R package. The ORA analysis only retained the top 300 genes obtained based on the median of HIRIPS.

**Statistical analysis**

Survival models were implemented via *R* packages including *mboost*, *survivalmodels*, *partykit*, *CoxBoost*, *gym*, *obliqueRSF*, *ranger*, *randomForestSRC*, *rpart*, *survivalsvm*, *xgboost*, *glmnet*, *plsRcox*, and *superpc*. Pearson or Spearman correlation coefficients were calculated to assess relationships between two variables. The Wilcoxon rank-sum test or T-test was used to compare continuous variables. The *survival* and *survminer* *R* packages conducted survival analysis. ROC analysis were performed using *timeROC* R packages, respectively. All data processing, statistical analysis, and plotting were realized in the *R* 4.1.2 environment.

1. Charoentong P, Finotello F, Angelova M, et al. Pan-cancer Immunogenomic Analyses Reveal Genotype-Immunophenotype Relationships and Predictors of Response to Checkpoint Blockade. Cell Rep 2017;18(1):248-262. DOI: 10.1016/j.celrep.2016.12.019.

2. Yoshihara K, Shahmoradgoli M, Martinez E, et al. Inferring tumour purity and stromal and immune cell admixture from expression data. Nat Commun 2013;4:2612. DOI: 10.1038/ncomms3612.

3. Yanai I, Benjamin H, Shmoish M, et al. Genome-wide midrange transcription profiles reveal expression level relationships in human tissue specification. Bioinformatics 2005;21(5):650-9. DOI: 10.1093/bioinformatics/bti042.

4. Sun J, Zhang Z, Bao S, et al. Identification of tumor immune infiltration-associated lncRNAs for improving prognosis and immunotherapy response of patients with non-small cell lung cancer. J Immunother Cancer 2020;8(1). DOI: 10.1136/jitc-2019-000110.

5. Liu Z, Guo C, Dang Q, et al. Integrative analysis from multi-center studies identities a consensus machine learning-derived lncRNA signature for stage II/III colorectal cancer. EBioMedicine 2022;75:103750. (In eng). DOI: 10.1016/j.ebiom.2021.103750.

6. Liu Z, Liu L, Weng S, et al. Machine learning-based integration develops an immune-derived lncRNA signature for improving outcomes in colorectal cancer. Nat Commun 2022;13(1):816. (In eng). DOI: 10.1038/s41467-022-28421-6.

7. Liu Z, Liu L, Weng S, et al. BEST: a web application for comprehensive biomarker exploration on large-scale data in solid tumors. bioRxiv 2022:2022.10.21.513300. DOI: 10.1101/2022.10.21.513300.
