# Supplementary material for: Implications of pseudogenes for the prognosis of hepatocellular carcinoma
Source: Clin Transl Med. 2023 Feb 7;13(2):e1195. doi: 10.1002/ctm2.1195 (PMC9905005; doi:10.1002/ctm2.1195)
Supplement: Supplementary file 2 — Supporting information [file CTM2-13-e1195-s001.docx]

Supplementary Figures

⚫        Figure S1

⚫        Figure S2


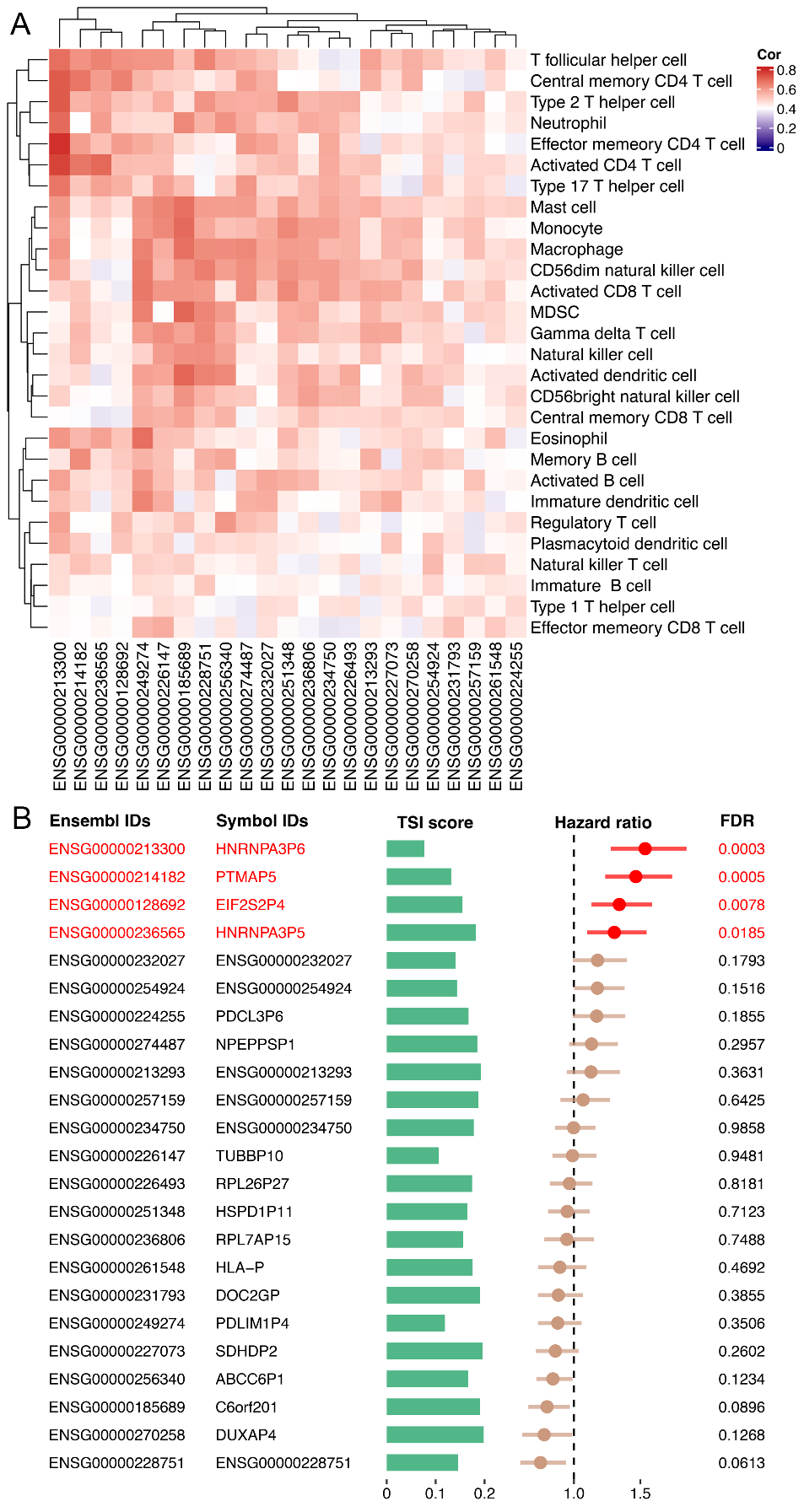


**Figure S1. Identification of tumor-infiltrating immune cell-associated** **pseudogenes.** (**A**) Heatmap showing the correlation between 23 pseudogenes and 28 immune cells. (**B**) Univariate Cox regression analysis of OS for the 23 pseudogenes in TCGA-LIHC. TSI: tissue specificity index, FDR: false discovery rate.


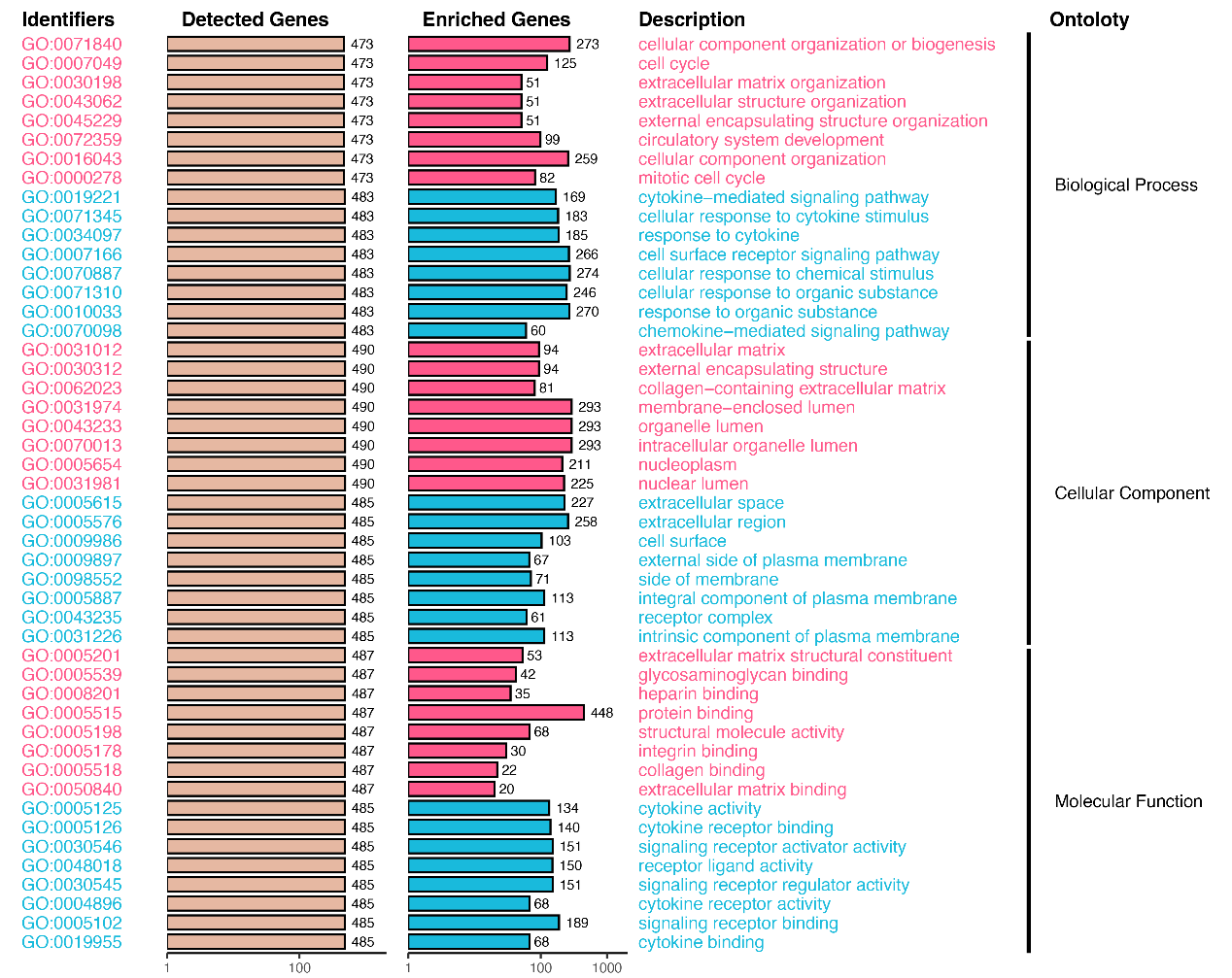


**Figure S2. Biological functions related to HIRIPS.** GO functional categories and terms that were significantly enriched in high HIRIPS are shown in red; those significantly enriched in low HIRIPS are shown in blue.
